# The Prognostic Value and Immunomodulatory Role of Spsb2, a Novel Immune Checkpoint Molecule, in Hepatocellular Carcinoma

**DOI:** 10.3390/genes16030346

**Published:** 2025-03-17

**Authors:** Lv Tian, Yiming Wang, Jiexin Guan, Lu Zhang, Jun Fan

**Affiliations:** 1 Institute of Fundamental and Frontier Sciences, University of Electronic Science and Technology of China, Chengdu 611731, China; 2 School of Nursing, Jilin University, Changchun 130021, China

**Keywords:** hepatocellular carcinoma, SPSB2, immune checkpoints, immune infiltration, prognosis, biomarkers

## Abstract

Background: Liver cancer, specifically hepatocellular carcinoma (LIHC), ranks as the second most common cause of cancer-related fatalities globally. Moreover, the occurrence rate of LIHC is steadily increasing. A recently identified gene, *SPSB2*, has been implicated in cell signaling, impacting the development and progression of non-small cell lung cancer. Nevertheless, studies on the role of SPSB2 in the pathogenesis of LIHC are lacking. Methods: Using the TCGA, GTEx, and GEO databases, we obtained differentially expressed genes that affect the prognosis of patients with LIHC. We utilized the Kruskal–Wallis test, along with univariate and multivariate COX regression analyses, to determine the correlation between SPSB2 and patient clinical indicators. Potential biological functions of SPSB2 in LIHC were explored by enrichment analysis, ssGSEA, and Spearman correlation analysis. Finally, LIHC cell lines Huh7 and SMMC-7721 were used to validate the biological function of SPSB2. Results: The results showed LIHC patients with higher SPSB2 expression had a poorer prognosis, and SPSB2 expression was significantly correlated with LIHC patients’ Histologic grade, Pathologic T stage, Prothrombin time, Pathologic stage, BMI, weight, adjacent hepatic tissue inflammation, AFP level, and OS event (*p* < 0.05). SPSB2 shows notable enrichment in pathways linked to tumorigenesis and the immune system. Moreover, its expression is strongly connected to immune cells and immune checkpoints. Knockdown of SPSB2 expression in Huh7 cells and SMMC-7721 cells inhibits SPSB2’s biological functions, including proliferation, invasion, metastasis, and other phenotypes. Conclusions: SPSB2 plays a crucial role in the development of LIHC. It is related to the immune response and unfavorable outcomes. SPSB2 may function as a clinical biomarker for prognosis.

## 1. Introduction

Hepatocellular carcinoma (LIHC) represents the predominant form of primary liver cancer, and LIHC is one of the second deadliest cancers worldwide [1]. Prominent risk factors contributing to hepatocellular carcinoma encompass chronic infections caused by the hepatitis B virus (HBV) and the hepatitis C virus (HCV) [2,3]. In addition, genetic factors, heavy alcohol intake, long-term smoking, and obesity are also important factors leading to LIHC [4]. Currently, treatments for hepatocellular carcinoma encompass surgery, liver transplantation, radiotherapy, and chemotherapy [5]. Nonetheless, the overall prognosis for hepatocellular carcinoma patients remains dismal due to several factors, such as cancer cells’ invasive and migratory properties, their resistance to drugs, and the lack of certainty in diagnosis [6]. In recent years, treatments for LIHC have begun to shift toward immunotherapy and novel targeted agents, and there is evidence that the prognosis of LIHC patients is expected to improve [7,8]. Research has indicated that immune checkpoint inhibitors (ICIs) can bring positive outcomes for advanced-stage hepatocellular carcinoma patients [9]. Therefore, a thorough exploration of the pathophysiology of HCC, along with the discovery of novel therapeutic targets and prognostic biomarkers, holds substantial clinical importance in improving the efficiency of immunotherapeutic agents and enhancing patients’ prognosis and quality of life [10].

SplA/Ryanodine Receptor Domain and SOCS Box Containing 2 (SPSB2) is a substrate for the cullin-5-RING-E3 ubiquitin ligase complex [11,12,13]. The complex regulates the proteasomal degradation of inducible nitric oxide synthase (iNOS) [13,14,15], which plays a crucial role in the immune system and defense against infections. SPSB2 binds to the N-terminal disordered region peptide of iNOS, KEEKDINNNVKKKT [13]. SPSB2 functions as a negative regulator, recruiting the E3 ubiquitin ligase complex and inducing the polyubiquitination of iNOS, which results in proteasomal degradation. Thus, it modulates the host response to infection [13,15]. It has been shown that SPSB2 deficiency leads to the prolonged expression of iNOS and increases NO production by macrophages, which enhances the killing of Leishmania parasites [13]. Meanwhile, by targeting NS5A for ubiquitination and degradation, SPSB2 impedes hepatitis C virus replication [16]. Another study found that SPSB2 is associated with the level of FeNO, which is closely related to airway obstruction [17]. SPSB2’s family member, SPSB1, interacts with TβRII through its SPRY structural domain and promotes the degradation of TβRII, thus negatively regulating TGF-β signaling and inhibiting tumor cell migration and invasion [18]. SPSB2 also regulates transforming growth factor-β receptor II (TβRII) [18], so it may impact the tumorigenesis and progression of tumors. Several studies have shown that SPSB2 is a target gene of ETV4 and is connected with tumor invasion and dependent growth [19], as well as dismal prognosis in non-small cell lung cancer [20]. Nevertheless, no published studies exist regarding the role of SPSB2 in hepatocarcinogenesis. As a result, this research aimed to explore the connection between SPSB2, immune cell infiltration, and the prognosis of patients with hepatocellular carcinoma. This exploration has the potential to offer a fundamental molecular foundation for the early noninvasive diagnosis and immunotherapy of hepatocellular carcinoma.

## 2. Materials and Methods

### 2.1. Data Sources and Preprocessing

We obtained the differential RNA-sequencing (RNAseq) expression data of SPSB2 across different types of cancers in TPM format from UCSC XENA (https://xenabrowser.net/datapages/ accessed on 5 October 2024). The TCGA (The Cancer Genome Atlas) and GTEx (Genotype-TissueGenotype-Tissue Expression) datasets were all processed consistently through the Toil process [21]. We retrieved the differential RNAseq expression data of SPSB2 from the TCGA (https://portal.gdc.cancer.gov/ accessed on 7 October 2024) LIHC project for unpaired and paired samples. The data came in the level 3 HTSeq—FPKM format. First, we took the RNAseq data initially in FPKM format, converted it into the TPM format (transcripts per million reads), and then applied a log2 transformation. After that, we used the data in TPM format for all subsequent analyses. The differential analysis data for SPSB2 in dataset GSE45267 [22,23] were acquired from the GEO database via the GEOquery package (version 2.54.1) [24]. To acquire these data, probes associated with multiple molecules were removed (when dealing with probes corresponding to identical molecules, only the probe featuring the highest signal value was retained). Subsequently, the data underwent further normalization using the normalize Between Arrays function of the limma package (version 3.42.2) [25]. R (version 4.2.1) was utilized for all statistical analyses and visualizations.

### 2.2. Differential Gene Expression Analysis and Correlation Analysis

We conducted gene differential analysis on the RNAseq data in level 3 HTSeq-Counts format that we sourced from the TCGA LIHC project. For this analysis, we used the DESeq2 package [26]. We employed the STAT package (version 3.6.3) to conduct a single-gene correlation analysis on the expression profile data presented in TPM format. SPSB2 was the target molecule in this analysis. Using the results of differential gene analysis, volcano plots were generated by applying a threshold of |log2(FC)| > 1 and p.adj < 0.05. We input these differentially expressed genes into the STRING database [27]. Next, through network analysis with Cytoscape software (version 3.8.0), we determined their protein–protein interaction (PPIs). After that, we utilized the MCODE plugin to identify the HUB genes. Finally, leveraging the outcomes of the single-gene correlation analysis, we separately extracted the top 15 genes with the highest correlations in descending order of the |Pearson value|. Subsequently, we used these genes to create a single-gene co-expression heat map for SPSB2. Both the volcano map and the co-expression heat maps were generated using the ggplot2 package (version 3.3.3).

### 2.3. Differential Expression Analysis of SPSB2

We utilized the Mann–Whitney U test (Wilcoxon rank-sum test) to assess the variations in SPSB2 expression profiles among different types of cancer. We applied the Shapiro–Wilk normality test to check the normality of SPSB2 expression data from paired samples, unpaired samples, and the GSE45267 dataset. To analyze the differences in the data of unpaired samples, we employed the independent samples *t*-test. The independent samples *t*-test was used to analyze the differences between the data in the unpaired samples. The paired samples *t*-test was used to analyze the differences in the data from the paired samples, and the Mann–Whitney U-test (Wilcoxon’s rank sum test) was used to analyze the ANOVA of the data from GSE45267. We used ggplot2 (version 3.3.3) to visualize all the above analyses. A result of (*p* < 0.05) was considered statistically significant.

### 2.4. Clinical Correlation Analysis and Survival Prognosis of SPSB2 Expression

We conducted statistical analysis on the survival data of LIHC patients with the Survminer software package (version 3.2-10). Then, to generate the Kaplan–Meier survival curves for the overall survival (OS) of LIHC patients, we visualized the analysis results using the Survminer software package (version 0.4.9). Subsequently, we grouped the LIHC patients according to clinicopathological factors such as age, gender, and weight and performed a generative analysis of the plotted Kaplan–Meier survival curves. We then calculated the correlations between these clinicopathological factors and SPSB2 expression. Finally, we used the ggplot2 package (version 3.3.3) to visualize the calculated results. We carried out an ROC analysis on the data with the pROC software package (version 1.17.0.1) to evaluate the accuracy of SPSB2 in prognostic prediction. Finally, we used the median to determine the threshold for SPSB2 expression. Again, we performed univariate and multivariate Cox regression analyses of clinicopathological factors and SPSB2 expression using the Survival software package (version 3.2-10). The results are visualized in a forest plot. The results are presented in forest plots for better visualization. All the predictive data used in the aforementioned survival analyses were sourced from a research paper published in *Cell* [28].

### 2.5. Functional Enrichment Analysis

We utilized the clusterProfiler package (version 3.14.3) to conduct GO, KEGG, and GSEA functional enrichment analyses on the outcomes of the gene differential analysis [29]. We used the org.Hs.eg.db package (version 3.10.0) to convert gene IDs. Then, we computed the Z-scores with the GOplot package (version 1.0.2). These scores are used to assess the correlation between SPSB2 and the enriched pathways [30]. For the GSEA, we employed the reference gene set c2.cp.all.v2022.1.Hs.symbols. gmt [All Canonical Pathways] [31]. An enrichment result was deemed significant when it satisfied two criteria: a false discovery rate (FDR) of less than 0.25 and an adjusted *p*-value (p.adjust) of less than 0.05. Afterwards, we used the ggplot2 package (version 3.3.3) to visualize the outcomes of the above analyses.

### 2.6. Immunoinfiltration Analysis

The relative infiltration levels of 24 immune cells were analyzed using the GSVA software package (version 1.34.0) [32]. The ssGSEA algorithm was employed to assess immune infiltration, with Spearman’s correlation analysis selected as the primary analytical approach. Twenty-four markers of immune cells were obtained from an immune-related study [33]. Subsequently, the samples were stratified into low- and high-expression cohorts according to their SPSB2 expression levels. The GSVA software package (version 1.34.0) was utilized to compute and evaluate the enrichment scores of diverse immune cell infiltrates across distinct subgroups. The relationship between SPSB2 and key immune checkpoint markers, including NFRSF4 (Neurotrophic Factor Receptor Superfamily 4), PDCD1 (Programmed Cell Death Protein 1), and LAG3 (Lymphocyte-activation Gene 3), was assessed through Spearman correlation analysis. The association between CD47 expression levels and CTLA4 (Cytotoxic T lymphocyte-Associated Protein 4) was also examined. Finally, the correlation between immune cell infiltration and SPSB2 expression levels was graphically represented through statistically significant immune cell infiltration data (*p* < 0.05). Utilizing SPSB2 gene expression profiles, chord diagrams were generated to illustrate these relationships. All statistical analysis and visualization were conducted employing the circlize package (version 0.4.12) [34].

### 2.7. Differential Analysis of SPSB2 Protein Expression Levels in LIHC

Immunohistochemical staining results demonstrating SPSB2 expression patterns in both hepatocellular carcinoma (LIHC) and normal hepatic tissues were acquired from the Human Protein Atlas database (https://www.proteinatlas.org/, accessed on 7 October 2024), followed by quantitative analysis of the obtained histological images.

### 2.8. Cell Lines and Culture

Two human hepatocellular carcinoma cell lines, Huh7 and SMMC-7721, were maintained in high-glucose DMEM medium (Sigma-Aldrich, St. Louis, MO, USA) supplemented with 10% fetal bovine serum (FBS; Excell Bio, Wuxi, China) and 1% penicillin-streptomycin (P/S; Solarbio Beijing, China). Cell cultivation was performed under standard conditions at 37 °C with 5% CO_2_ in a humidified incubator.

### 2.9. Cell Transfection

We procured small interfering RNA (siRNA) oligonucleotides designed to target SPSB2 and control non-specific siRNA from Thermo Fisher (Waltham, MD, USA) in the United States. We used the jetPRIME (#150-15) transfection reagent from Polyplus in France for transient transfections. The transfection process was carried out strictly according to the manufacturer’s guidelines.

### 2.10. Real-Time-PCR

Following PBS rinsing, cellular lysates were prepared by adding Trizol reagent and chloroform, with mechanical homogenization facilitating phase separation. After 10 min of incubation at ambient temperature, samples underwent centrifugation (12,000× *g*, 15 min, 4 °C). The aqueous phase was aliquoted into fresh microcentrifuge tubes and combined with isopropanol (1:1 *v*/*v*). Subsequent centrifugation yielded RNA pellets that underwent 75% ethanol purification, followed by dissolution in RNase-free water post-desiccation. RNA quantification was conducted spectrophotometrically.

Reverse transcription was performed using the TransGen Biotech (Beijing, China) kit per the manufacturer’s protocol. Quantitative PCR analysis employing SYBR Green I chemistry implemented thermal cycling parameters: initial denaturation (94 °C, 30 s); 40 cycles of denaturation (94 °C, 5 s), and annealing/extension (60 °C, 30 s). Gene expression quantification utilized the 2^−ΔΔCt^ method, with β-actin as endogenous control. Oligonucleotide primers (Sangon Biotech, Shanghai, China) were designed as

β-Actin: F 5′-CCTGGCACCCAGCACAAT-3′, R 5′-GGGCCGGACTCGTCATAC-3′

SPSB2: F 5′-CCGGGGTAAGAGGGGCTATT-3′, R 5′-CCGCGTAGTGGTCAGTCTG-3′

### 2.11. Cell Proliferation Assay

Cellular proliferation was evaluated utilizing the Cell Counting Kit-8 (CCK-8; Catalog B34304, Selleckchem). Briefly, cells were seeded in 96-well plates at a density of 4 × 10^3^ cells/well and cultured under standardized conditions (37 °C, 5% CO_2_). At predetermined time intervals (0, 24, 48, and 96 h post-seeding), 10 μL of CCK-8 reagent was aliquoted into each well. Following a 2 h incubation period, optical density measurements were obtained at 450 nm wavelength using a microplate reader.

### 2.12. Colony Formation Assay

We seeded 1000–1500 cells per well in six-well plates. After subjecting the cells to the specified treatments, we let them grow for 7 days. Subsequently, we fixed the cells using 4% paraformaldehyde (product code P1110, sourced from Solarbio Beijing, China) and then stained them with 5% crystal violet (product code G1063, sourced from Solarbio Beijing, China). The number of visible colonies was counted to assess the colony-forming ability of the cells. All experiments were repeated three times.

### 2.13. Wound-Healing Assay

We plated cells in six-well plates at a density of 3 × 10⁵ cells per well. Then, we used a 200 μL pipette tip to create a straight scratch on the cell monolayer, forming a linear wound. After that, the cells were incubated in a serum-free culture medium at 37 °C with 5% CO_2_. We continued the cultivation for an extra 24 and 48 h, respectively. To monitor the migration of cells into the wound area at the 24 h and 48 h time points, we captured images using a light microscope (manufactured by Motic Corporation Xiamen, China) equipped with a digital camera. All the micrographs were taken at the same magnification and at consistent time intervals to ensure comparability.

### 2.14. Transwell Migration Assay

We suspended 2.5 × 10^5^ cells in 1.5 mL of serum-free medium and added this cell suspension to the upper chamber of a 6-well Transwell plate (3428, from Corning, NY, USA). Simultaneously, 2.6 mL of medium supplemented with 10% fetal bovine serum (FBS) was placed in the bottom chamber. The Transwell plate was then incubated at 37 °C for 48 h. Post-incubation, the cells were washed twice with phosphate-buffered saline (PBS). Next, they were fixed using 4% paraformaldehyde and stained with crystal violet. Finally, we counted the number of migrating cells in three fields of view for all the chambers.

### 2.15. Statistical Analysis

The data are presented as the mean plus or minus the standard deviation (mean ± SD). To assess the disparity in SPSB2 expression between LIHC tumor tissues and normal tissues, we employed a Student’s *t*-test. When comparing multiple groups, we used a one-way analysis of variance (ANOVA). We utilized the Mann–Whitney U test to examine the association between SPSB2 expression and the clinical data of patients with LIHC. We created the statistical graphs with GraphPad Prism 8. A result was considered statistically significant if the *p*-value was less than 0.05. We computed the Spearman correlation coefficient to gauge the correlation between SPSB2 and other genes.

## 3. Results

### 3.1. Differential Gene Expression Analysis and Correlation Analysis of SPSB2

**Figure 1:**
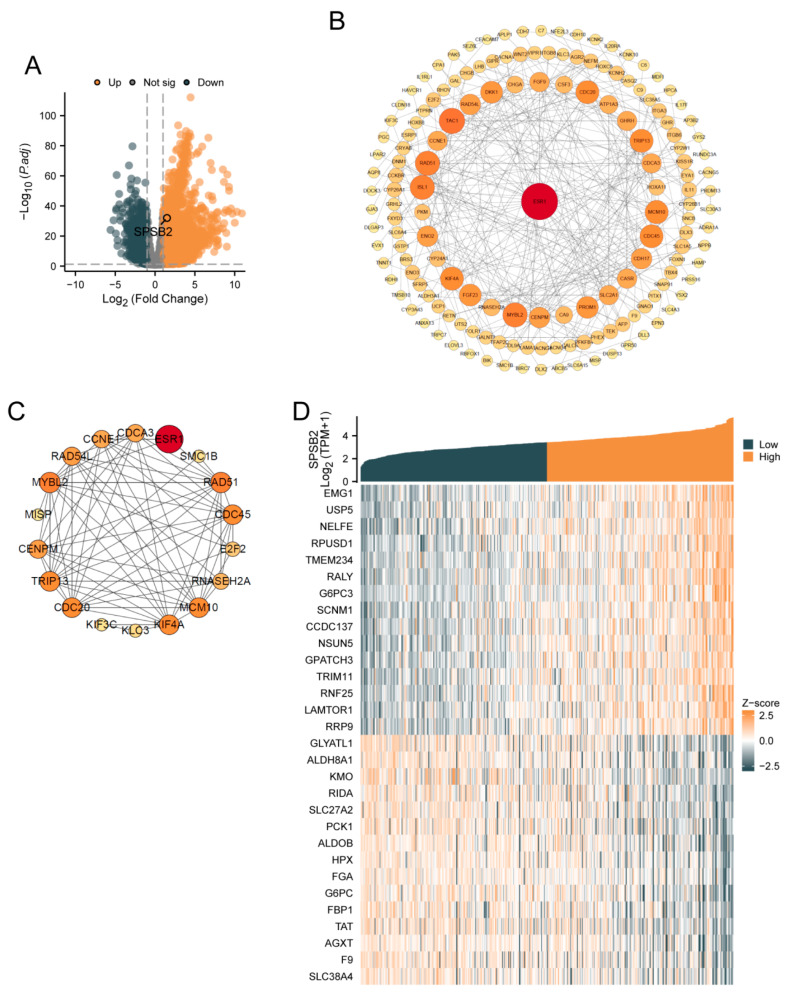
Gene differential analysis and correlation analysis of SPSB2 in LIHC. (**A**) Volcano plot for the differential analysis of the single gene SPSB2. (**B**) Protein–protein interaction network (PPI) of the differentially expressed genes in the differential analysis of the single gene SPSB2. (**C**) Protein–protein interaction network diagram (PPI) of the HUB genes. (**D**) Co-expression heatmap of the top 15 genes with positive and negative correlations to SPSB2 in the single gene correlation analysis.

SPSB2 was genetically differentially analyzed in LIHC. Among the genes that met the threshold of |log2(FC)| > 1 and p.adj < 0.05, there were a total of 9457 genes (Figure 1A). Under this threshold, 3229 genes showed high expression of the encoded protein (log2FC > 0), and 1232 genes showed low expression of the encoded protein (log2FC < 0). After excluding 3295 genes that were not related to prognosis, a PPI network was constructed using 1236 differentially expressed genes. This PPI network is shown in Figure 1B. The nearer the genes are to the center of the interaction network diagram, the greater the connections they establish with other genes. Subsequently, we employed the MCODE plugin to identify 18 hub genes: ESR1, MYBL2, SMC1B, RNASEH2A, KIF4A, E2F2, RAD51, KIF3C, CCNE1, RAD54L, KLC3, CDC20, TRIP13, CDCA3, MCM10, CDC45, MISP, and CENPM. The PPI network of HUB genes is shown in Figure 1C. Finally, single-gene correlation analysis was performed for SPSB2, and the top 15 genes with the strongest positive and negative correlations were selected. Co-expression heatmaps with SPSB2 were plotted, and the results are shown in Figure 1D.

### 3.2. Differential Expression of SPSB2 in Pancancer and LIHC

**Figure 2:**
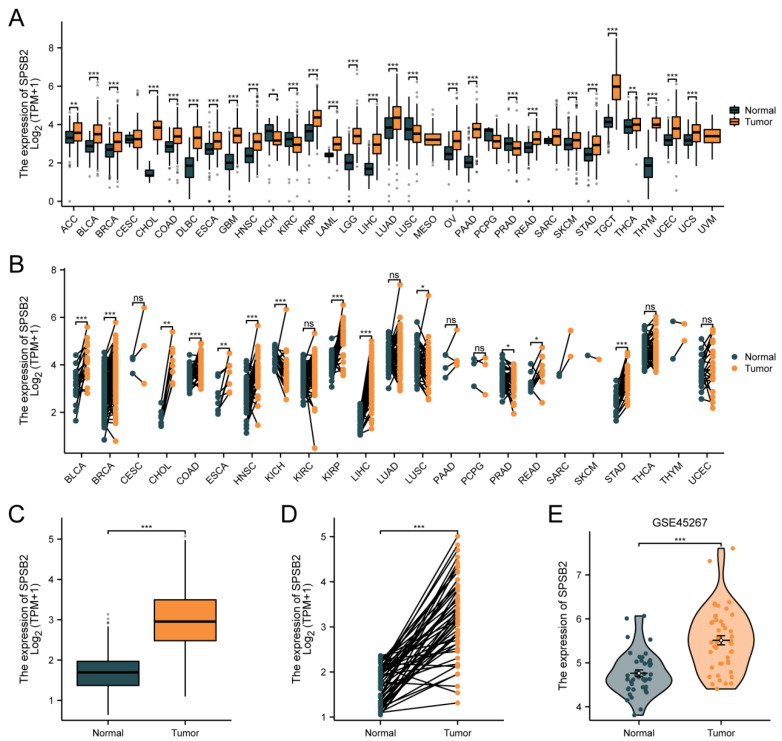
Differential expression of SPSB2 in pan-cancer and LIHC. (**A**) Differential expression of SPSB2 in 33 types of tumors within the TCGA database. (**B**) Differential expression of SPSB2 in various paired tumors within the TCGA database. (**C**) Differential expression of SPSB2 in unpaired samples of LIHC. (**D**) Differential expression of SPSB2 in paired samples of LIHC. (**E**) Differential expression of SPSB2 in the GSE45267 dataset. Significance identifier: ns (no significance, *p* ≥ 0.05); * *p* < 0.05; ** *p* < 0.01; *** *p* < 0.001.

Figure 2A depicts differential SPSB2 expression patterns across malignancies. Significant downregulation (*p* < 0.05) was observed in kidney chromophobe (*n* = 66 tumor vs. 53 normal), renal clear cell carcinoma (531 vs. 100), lung squamous cell carcinoma (498 vs. 338), and prostate adenocarcinoma (496 vs. 152) compared to matched controls. Conversely, marked overexpression (*p* < 0.05) emerged in adrenocortical carcinoma (77 vs. 128), bladder urothelial carcinoma (407 vs. 28), breast invasive carcinoma (1099 vs. 292), cholangiocarcinoma (36 vs. 9), colon adenocarcinoma (290 vs. 349), diffuse large B-cell lymphoma (47 vs. 444), oesophagal carcinoma (182 vs. 666), glioblastoma (166 vs. 1157), head–neck squamous carcinoma (520 vs. 44), renal papillary carcinoma (289 vs. 60), acute myeloid leukemia (173 vs. 70), low-grade glioma (523 vs. 1152), hepatocellular carcinoma (371 vs. 160), lung adenocarcinoma (515 vs. 347), ovarian serous cystadenocarcinoma (427 vs. 88), pancreatic adenocarcinoma (179 vs. 171), cutaneous melanoma (469 vs. 813), rectal adenocarcinoma (93 vs. 318), gastric adenocarcinoma (414 vs. 210), testicular germ cell tumors (154 vs. 165), thyroid carcinoma (512 vs. 338), thymoma (119 vs. 446), endometrial carcinoma (181 vs. 101), and uterine carcinosarcoma (57 vs. 78). As shown in Figure 2B, in pan-cancer pairs, the expression of SPSB2 in BLCA (T = 19, N = 19), BRCA (T = 133, N = 133), CHOL (T = 8, N = 8), COAD (T = 41, N = 41), ESCA (T = 8, N = 8), HNSC (T = 43, N = 43), KIRP (T = 32, N = 32), LIHC (T = 50, N = 50), READ (T = 9, N = 9), STAD (T = 27, N = 27) was significantly higher than in the adjacent tissue (*p* < 0.05). In both paired and unpaired LIHC samples, the expression of SPSB2 was significantly different compared to normal samples (Figure 2C,D). Subsequently, we utilized the GSE45267 dataset from the GEO database to verify the findings obtained from the TCGA database. As depicted in Figure 2E, the differences in the results remained significant.

### 3.3. Correlation of SPSB2 Expression with Clinicopathologic Parameters

**Figure 3:**
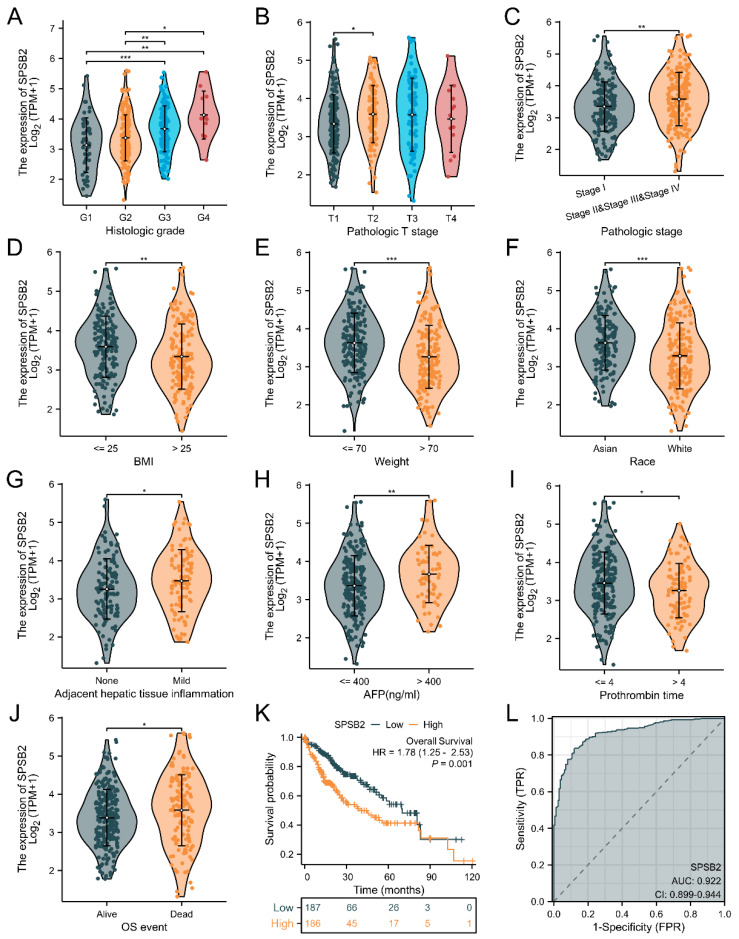
Clinical correlation analysis of SPSB2 expression. (**A**–**J**) Expression of SPSB2 in different patient groups with diverse clinicopathological factors. (**K**) Kaplan–Meier survival curve demonstrating the relationship between SPSB2 expression and overall survival (OS). (**L**) ROC curve. The area under the ROC curve (AUC) ranges from 0.5 to 1. An AUC approaching 1 denotes superior diagnostic performance. An AUC in the range of 0.5–0.7 indicates low accuracy, 0.7–0.9 represents moderate accuracy, and an AUC above 0.9 implies high accuracy. Significance identifier: * *p* < 0.05; ** *p* < 0.01; *** *p* < 0.001.

We explored the association between SPSB2 expression and the diverse clinical features of LIHC patients. To examine the correlation between clinicopathological factors and SPSB2 expression, we employed the Chisq test along with Yates’ correction. The detailed results are presented in Table 1. The Chisq test showed that weight (*p* < 0.001), Race (*p* < 0.001), Pathologic T stage (*p* = 0.017), Pathologic stage (*p* = 0.041), BMI (*p* < 0.001), Histologic grade (*p* < 0.001), and level of AFP (*p* = 0.024) were correlated. As shown in Figure 3A–J, based on the Kruskal–Wallis test and Dunn’s test, SPSB2 expression was correlated with Histologic grade, Pathologic T stage, Pathologic stage, BMI, weight, Race, adjacent hepatic tissue inflammation, AFP level, Prothrombin time, and OS event (*p* < 0.05). Survival analysis (Figure 3K) revealed that in LIHC patients, high SPSB2 expression was linked to worse overall survival (OS). An area under the curve (AUC) of 0.922 indicated that SPSB2 could serve as a diagnostic biomarker (Figure 3L).

### 3.4. Subgroup Analysis of Survival Prognosis of SPSB2 Expression

**Figure 4:**
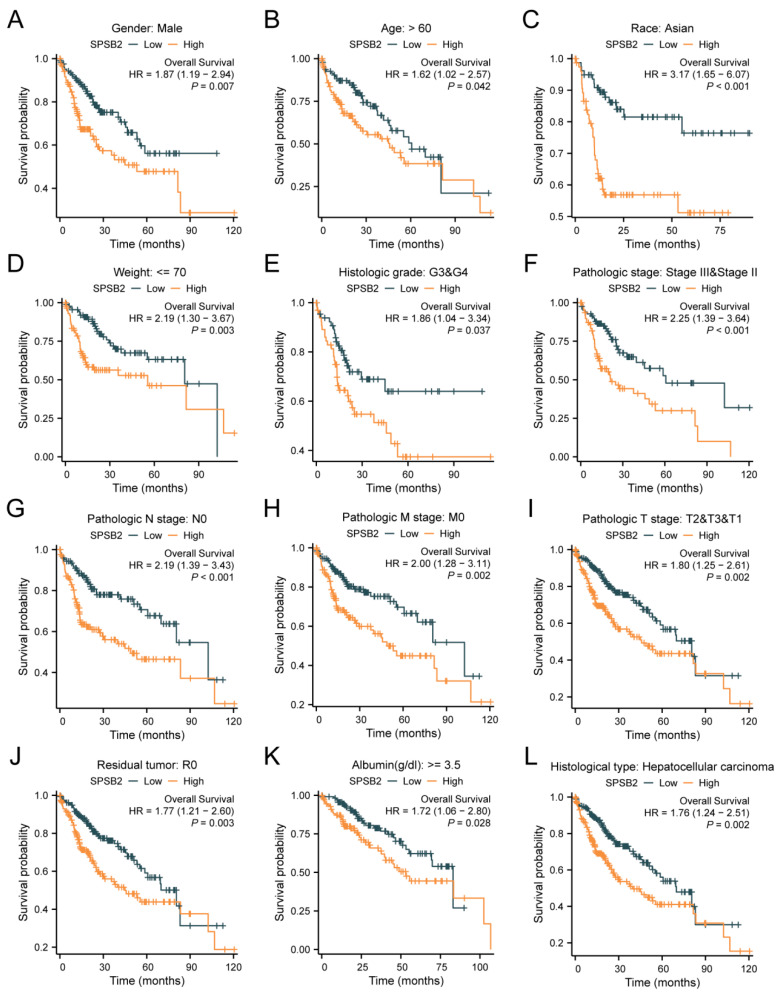
Subgroup prognostic analysis of survival and SPSB2 expression. (**A**–**L**) Kaplan–Meier survival curves depict the relationship between SPSB2 expression and overall survival (OS) in different patient subgroups with diverse clinicopathological factors.

Figure 4A–L shows the survival of patients with high or low SPSB2 expression in different LIHC subgroups. The results show the subgroups with male gender (HR = 1.87 (1.19–2.94), *p* = 0.007), subgroups with age over 60 years (HR = 1.62 (1.02–2.57), *p* = 0.042), subgroups with ethnicity Asian (HR = 3.17 (1.65–6.07), *p* < 0.001), subgroups with weight less than 70 kg (HR = 3.17 (1.65–6.07), *p* < 0.001), and subgroups with weight less than 70 kg (HR = 3.17 (1.65–6.07), *p* < 0.001). Subgroups with body weight less than 70 kg (HR = 2.19 (1.30–3.67), *p* = 0.003), subgroups with Histologic grade staging of G3&G4 (HR = 1.86 (1.04–3.34), *p* = 0.037), Pathologic stage staging as the subgroup of Stage III and Stage II (HR = 2.25 (1.39–3.64), *p* < 0.001), Pathologic N stage staging as the subgroup of Stage N0 (HR = 2.19 (1.39–3.43), *p* < 0.001), Pathologic M stage staging as a subgroup of M0 staging (HR = 2.00 (1.28–3.11), *p* = 0.002), Pathologic T stage staging as a subgroup of T2&T3&T1 (HR = 1.80 (1.25–2.61), *p* = 0.002), subgroups with a Residual tumor of R0 (HR = 1.77 (1.21–2.60), *p* = 0.003), subgroups with Albumin level ≥ 3.5 (HR = 1.72 (1.06–2.80), *p* = 0.028), and the subgroups with Histological type of Hepatocellular carcinoma (HR = 1.76 (1.24–2.51), *p* = 0.002) were associated with increased SPSB2 expression and low overall survival. To examine the effects of SPSB2 expression and clinicopathologic parameters on survival, we used both one-way and multifactorial Cox regression analyses (Table 2). Among the variables with *p* < 0.05 in the univariate Cox regression model, Pathologic T stage, Pathologic M stage, and SPSB2 expression level were statistically significant. Then, the multivariate Cox regression model included these variables as well as ULBP2. In this study, the Pathologic T3 stage (*p* < 0.001), Pathologic T3 stage (*p* = 0.002), and SPSB2 (*p* = 0.004) independently affected the overall survival of LIHC patients.

### 3.5. Functional Enrichment Analysis of SPSB2 in LIHC

**Figure 5:**
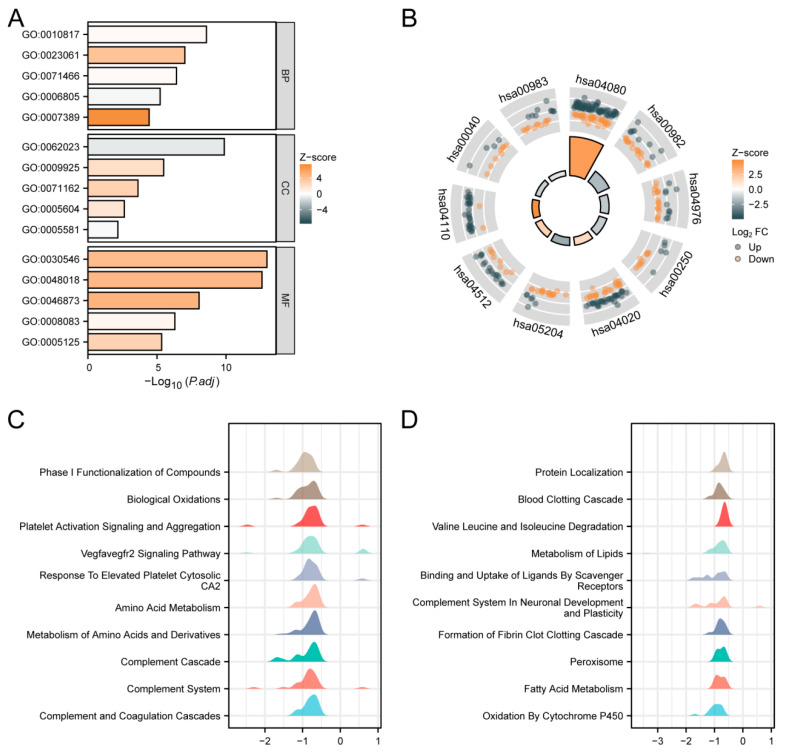
Functional enrichment analysis of SPSB2 in LIHC. (**A**) Results of GO analysis. (**B**) KEGG analysis results. (**C**,**D**) Results of GSEA. When the abscissa is positive, it indicates a positive correlation between SPSB2 expression and the pathway; when it is negative, the opposite is true.

We conducted GO, KEGG, and GSEA enrichment analyses using the outcomes of single-gene differential analysis. The results of these analyses are presented in Figure 5. Figure 5A and Table 3 display the findings of the GO analysis, showing that in the biological process (BP) category, SPSB2 regulates hormone levels, signal release, cellular response to xenobiotic stimuli, xenobiotic metabolic processes, and pattern specification processes. In the cellular component (CC) category, SPSB2 is associated with collagen-containing extracellular matrix, basal plasma membrane, CMG complex, basement membrane, and collagen trimer. Within the molecular function (MF) category, SPSB2 is found to be related to signaling receptor activator activity, receptor–ligand activity, metal ion transmembrane transporter activity, growth factor activity, and cytokine activity. Figure 5B and Table 4 illustrate the results of the KEGG analysis, demonstrating that SPSB2 is associated with various signaling pathways, including Neuroactive ligand-receptor interaction, drug metabolism—cytochrome P450, bile secretion, Alanine, aspartate, and glutamate metabolism, calcium signaling pathway, Chemical carcinogenesis—DNA adducts, ECM-receptor interaction, cell cycle, Pentose and glucuronate interconversions, and drug metabolism—other enzymes. The Z-scores reflect the degree of correlation between SPSB2 and these pathways. If the Z-score is negative, it shows a negative correlation, and when it is positive, it shows a positive correlation. Figure 5C,D present the results of GSEA, showing significant enrichment of genes related to tumor occurrences, invasions, and angiogenesis, such as Phase I Functionalization of Compounds, Biological Oxidations, Platelet Activation Signaling and Aggregation, Vegfavegfr2 Signaling Pathway, response to elevated Platelet Cytosolic Ca2, amino acid metabolism, metabolism of amino acids and derivatives, Complement Cascade, Complement System, Complement and Coagulation Cascades, and others.

### 3.6. Immunoinfiltration Analysis of SPSB2

**Figure 6:**
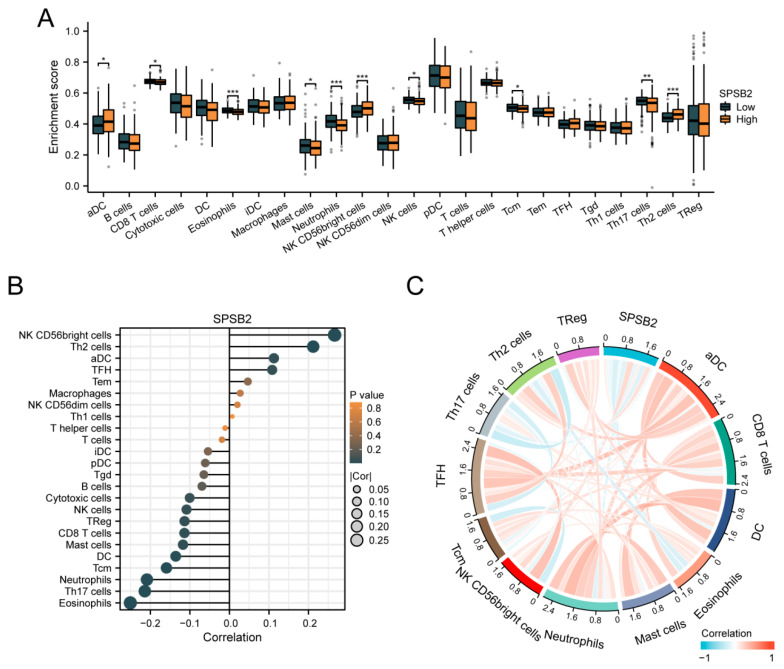
Immune infiltration analysis of SPSB2. (**A**) Group comparison of the infiltration levels of 24 immune cell types between the low SPSB2-expressing group and the high SPSB2-expressing group. (**B**) Results of the correlation between SPSB2 expression and 24 immune cell types. (**C**) Chord diagram of the correlations between SPSB2 and the infiltration levels of multiple immune cell types. The darker the color, the stronger the correlation. Red indicates a positive correlation, and dark blue indicates a negative correlation. Significance identifier: * *p* < 0.05; ** *p* < 0.01; *** *p* < 0.001.

To assess the impact of SPSB2 on the tumor microenvironment, we employed the ssGSEA method for immune infiltration analysis. We calculated the correlation between the enrichment of immune cells and the expression levels of SPSB2 in LIHC tissues through Spearman correlation analysis (Figure 6B). The expression of SPSB2 exhibited a positive correlation with the infiltration levels of four kinds of immune cells: activated dendritic cells (aDCs), CD56-bright natural killer (NK CD56bright) cells, follicular helper T (TFH) cells, and T-helper 2 (Th2) cells. The expression of SPSB2 was negatively associated with the infiltration level of nine types of immune cells: Eosinophils, Th17 cells, neutrophils, Tcm, DC, Mast cells, CD8 T cells, Treg, and NK cells. Subsequently, we classified the expression profile data into high and low expression groups according to the expression level of SPSB2, detecting the variations in the infiltration levels of immune cells among different groups. The results (Figure 6A) revealed that for aDCs, NK CD56bright cells, and Th2 cells, the infiltration level in the low-expression group was notably lower than in the high-expression group. Conversely, regarding Eosinophils, Th17 cells, neutrophils, Tcm, DC, Mast cells, CD8 T cells, and NK cells, the infiltration level in the low-expression group was significantly higher than in the high-expression group. This outcome is in line with the results presented in Figure 6B. Finally, we plotted the expression values of SPSB2 against the enrichment scores of 12 immune cells with significant correlation as a chord plot to visualize the correlation between them, and the results are shown in Figure 6C.

### 3.7. Correlation Between SPSB2 Expression and Immune Checkpoints

**Figure 7:**
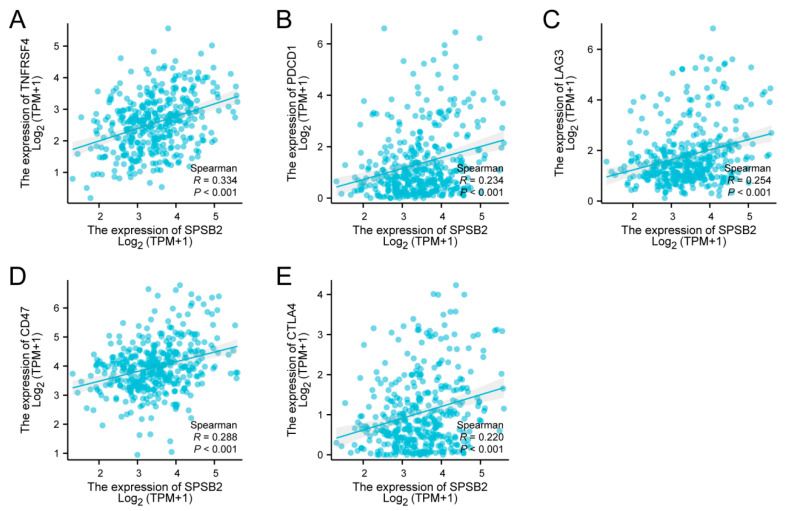
Tumor immune checkpoints and SPSB2 expression. (**A**–**E**) SPSB2 was significantly correlated with immune checkpoints TNFRSF4, PDCD1, LAG3, CD47, and CTLA4.

Spearman correlation analysis revealed significant positive associations between SPSB2 expression levels and multiple immune checkpoint markers: TNFRSF4 (ρ = 0.334, *p* < 0.001), PD-1 (ρ = 0.234, *p* < 0.001), LAG-3 (ρ = 0.254, *p* < 0.001), CD47 (ρ = 0.288, *p* < 0.001), and CTLA-4 (ρ = 0.220, *p* < 0.001) (Figure 7A–E).

### 3.8. Evaluation of SPSB2 Expression

**Figure 8:**
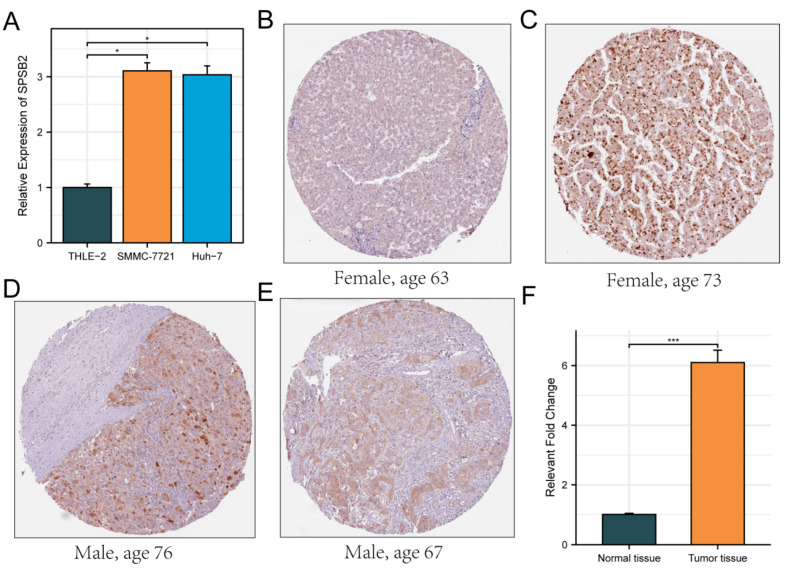
Evaluation of the expression of SPSB2 in LIHC cell line. (**A**) qPCR was employed to detect the expression of SPSB2 in the hepatocellular carcinoma cell lines SMMC-7721, Huh-7, and the normal liver cell line THLE-2. (**B**–**E**) Immunohistochemical images of SPSB2 protein expression in normal tissues (**B**) and LIHC (**C**–**E**) in the Human Protein Atlas (HPA) data. (**F**) Quantitative analysis of immunohistochemical images. Significance identifier: * *p* < 0.05; *** *p* < 0.001.

The Huh7 and SMMC-7721 are two commonly used human liver cancer cell lines. The Huh7 cell line was established from a well-differentiated hepatocellular carcinoma. It has characteristics such as high proliferative ability and a relatively stable genetic background, which makes it suitable for studying the basic biological functions of LIHC cells [35]. The SMMC-7721 cell line is also derived from hepatocellular carcinoma and has distinct features in terms of cell morphology and growth patterns. It shows a relatively high degree of malignancy and metastatic potential in some in vitro assays [36]. By using these two cell lines, we can more comprehensively investigate the biological function of SPSB2 in LIHC cells. Therefore, we evaluated the expression of SPSB2 in the LIHC cell lines SMMC-7721, Huh-7, and the normal liver cell line THLE-2 using real-time PCR. As shown in Figure 8A, the expression of SPSB2 was higher in hepatocellular carcinoma cells than in normal hepatocytes. In addition, the immunohistochemical results in the HPA database confirmed this finding (Figure 8B–F).

### 3.9. Effect of Knockdown of SPSB2 on the Biological Function of Hepatocellular Carcinoma Cells

**Figure 9:**
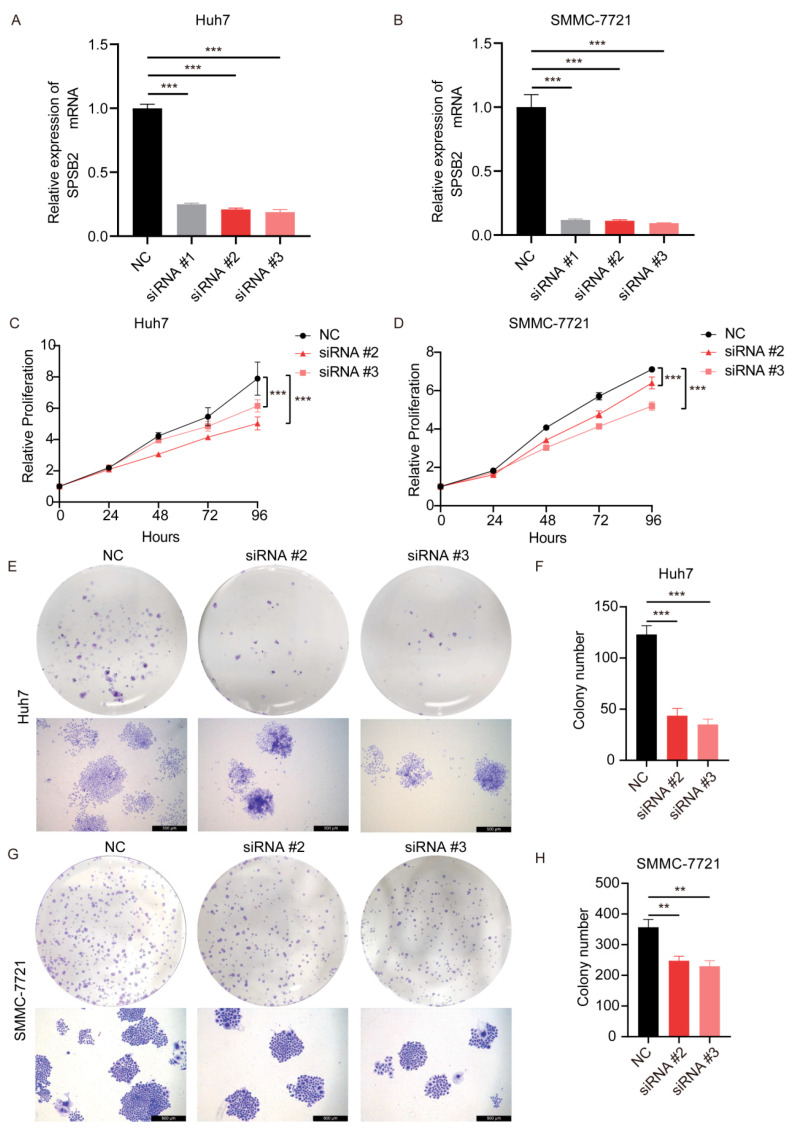
Effect of knockdown of SPSB2 expression in LIHC cell lines on tumor cell proliferation. (**A**,**B**) qPCR was utilized to detect the expression of the SPSB2 gene in Huh7 and SMMC-7721 cells following transfection with SPSB2-targeting siRNA. (**C**,**D**) The Cell Counting Kit—8 (CCK—8) assay was employed to assess the proliferative capacity of cells in the SPSB2 knockdown group and the control group at different time points. (**E**,**F**) Cell colony formation assay and quantitative analysis in Huh7 cells. (**G**,**H**) Cell colony formation assay and quantitative analysis in SMMC-7721 cells. Significance identifier: ** *p* < 0.01; *** *p* < 0.001.

**Figure 10:**
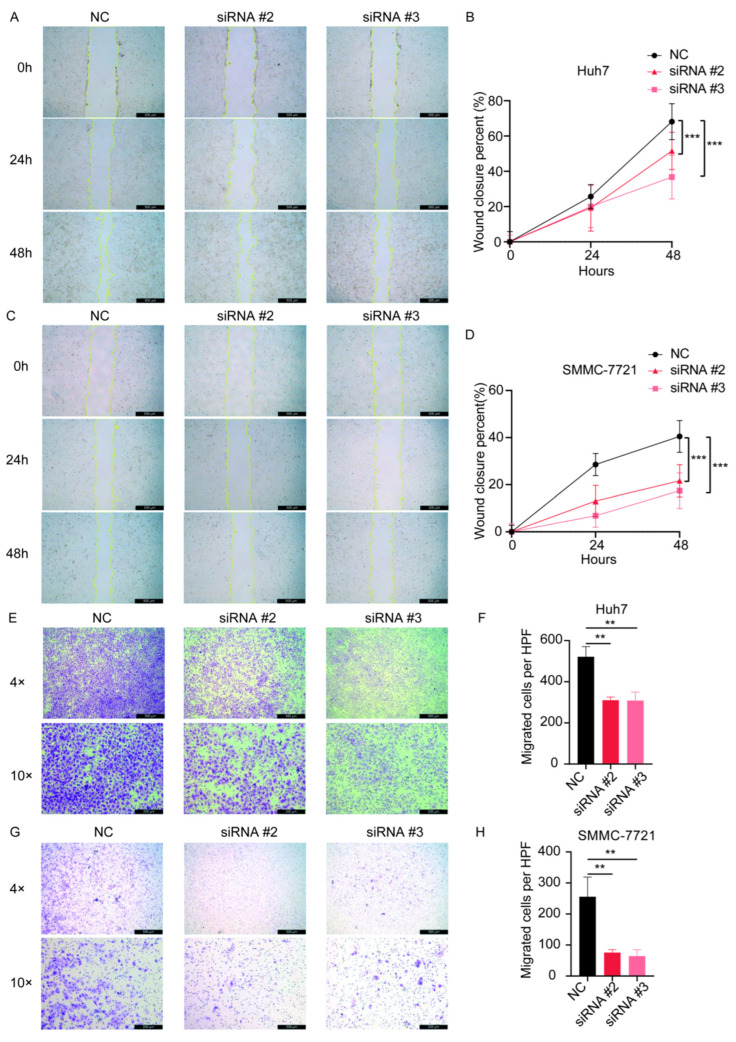
The effect of the knockdown of SPSB2 expression in LIHC cell lines on tumor cell wound-healing assay and Transwell migration assay. (**A**,**B**) Wound healing assay and quantitative analysis in Huh7 cells. (**C**,**D**) Wound healing assay and quantitative analysis in SMMC-7721 cells. (**E**,**F**) Transwell migration assay and quantitative analysis in Huh7 cells. (**G**,**H**) Transwell migration assay and quantitative analysis in SMMC-7721 cells. Significance identifier: ** *p* < 0.01; *** *p* < 0.001.

After transfection of the siRNAs, we examined the expression of the gene SPSB2, and the results showed that all three siRNAs could effectively inhibit the expression of SPSB2 in Huh7 and SMMC-7721 (Figure 9A,B), of which siRNA#2 and siRNA#3 were more efficiently knocked down and used for subsequent experiments. Subsequently, we performed cell proliferation assays (Figure 9C,D) and clone formation assays (Figure 9E–H), which showed that the proliferation ability of cells in the SPSB2 knockdown group was significantly lower than that of cells in the control group. We carried out both a wound-healing assay (Figure 10A–D) and a Transwell migration assay (Figure 10E–H). The findings from the wound-healing assay indicated that as the expression of SPSB2 declined, the metastatic capabilities of the two cell types were attenuated; the results of Transwell migration assay results showed that the invasion ability of cells in the SPSB2 knockdown group was also significantly weakened.

## 4. Discussion

Hepatocellular carcinoma (HCC) is the sixth most common cancer and the fourth leading cause of cancer-related deaths worldwide [37]. The 5-year survival rate for LIHC is only 15%, and its incidence and mortality are increasing every year. It is projected that by 2030, LIHC will rank as the third leading cause of cancer-related fatalities [38]. Since LIHC is frequently diagnosed at an advanced stage, the recurrence rate following tumor resection remains high, and the success rate of liver transplantation is low. Patients are prone to drug resistance and frequent recurrent metastases during treatment, making LIHC one of the tumors with the worst prognosis [39,40]. In the tumor microenvironment, interactions between cancer cells and host immune responses can promote or inhibit the pathological progression of cancer [41]. In recent years, novel therapeutic approaches like tumor immunosuppressive therapy have extended patients’ survival. Moreover, the combination of immune checkpoint inhibitors (ICIs) and vascular endothelial growth factor (VEGF) inhibitors has emerged as the first-line treatment option for advanced LIHC [40]. Immunotherapy is of great significance in managing LIHC. Hence, thoroughly comprehending the molecular mechanisms governing the progression of LIHC, exploring novel diagnostic and prognostic biomarkers and therapeutic targets, and formulating new anti-tumor drugs and immunotherapeutic approaches are indispensable means to enhance the survival rate of LIHC patients.

This research retrieved the clinical and RNA data of LIHC patients from the TCGA database. Subsequently, we used the “limma” and “survival” packages as well as Venn overlap analysis to obtain differentially expressed protein-coding genes associated with LIHC patients and prognosis and finally selected SPSB2 as a target gene. The analysis of the UCSC XENA and TCGA databases found that in LIHC, the expression level of SPSB2 was notably higher than that in normal liver tissue. This elevated SPSB2 expression was linked to a poor prognosis for patients. Specifically, the overall survival of LIHC patients with high-level SPSB2 expression was shorter compared to those with low-level SPSB2 expression. These findings imply that SPSB2 is related to the progression and prognosis of LIHC. In addition, SPSB2 expression was significantly associated with Histologic grade, Pathologic T stage, Pathologic stage, BMI, Weight, Race, Adjacent hepatic tissue inflammation, AFP level, Prothrombin time, and OS event, suggesting that SPSB2 may play an essential role in the biological function of tumor cells. These results suggest that SPSB2 may be a useful diagnostic molecular marker for LIHC and may predict the prognosis of LIHC patients. Diagnosis based on ROC curves (AUC = 0.922) and univariate and multivariate COX regression analyses further indicated that SPSB2 could be used for the diagnosis of LIHC. Thus, SPSB2 holds promise as a novel prognostic indicator, offering potential clinical utility in both diagnostic evaluation and outcome prediction for LIHC patients.

We carried out single-gene correlation analyses to delve deeper into predicting the molecular mechanism through which SPSB2 propels the development of LIHC. We established a protein–protein interaction (PPI) network. The outcomes demonstrated that the 18 hub genes most closely related to SPSB2 expression could predict the functions of SPSB2 genes. Among them, ESR1 and SPSB2 are particularly closely related. ESR1 encodes a transcription factor for estrogen receptor and ligand activation [42]. Research has discovered that the abnormal expression of the ESR1 gene is strongly linked to the prognosis of breast cancer [43,44,45]. Furthermore, the overexpression of ESR1 has been connected to the development and advancement of several other cancer types, including gastric cancer, prostate cancer, colorectal cancer, and lung cancer [46,47,48,49,50]. Therefore, we hypothesized that SPSB2 could potentially influence the development of LIHC through its modulation of ESR1. Among the genes closely related to SPSB2 expression, CDC45, RAD51, TRIP13, MYBL2, and CDC20 play essential roles in cancer development [51,52,53,54,55]. They are involved in processes such as cell cycle regulation and DNA repair, and aberrant expression or functional defects, which may lead to the accumulation of DNA damage, chromosomal instability, and abnormal cell proliferation, thereby promoting cancer development and progression. Our research indicates that elevated SPSB2 expression might be linked to gene mutations and tumor proliferation, invasion, and metastasis in patients with LIHC.

To gain a deeper insight into the molecular mechanism of SPSB2 in tumor initiation and progression, we conducted GO, KEGG, and GSEA functional enrichment analyses with SPSB2 and its associated differentially expressed genes. GO enrichment analysis revealed that SPSB2 and its co-expressed mRNAs were significantly enriched in multiple molecular functions. These included signaling receptor activator activity, receptor-ligand activity, metal ion transmembrane transporter activity, growth factor activity, and cytokine activity. These aberrant molecular functional activities may lead to the occurrence of malignant behaviors such as cell proliferation, survival, and migration and promote the development of cancer [56,57,58,59,60].

Moreover, KEGG analysis showed that the function of SPSB2 was associated with signaling pathways such as ECM-receptor interaction, cell cycle, calcium signaling pathway, drug metabolism—cytochrome P450, and bile secretion. The extracellular matrix interacts with receptors on the cell membrane and participates in biological processes such as cell adhesion, migration, proliferation, and differentiation by regulating the synthesis, degradation, and remodeling of the extracellular matrix, thereby affecting tumor cell growth and metastasis [61]. Cell cycle-related signaling pathways are involved in multiple cancer types, such as breast cancer, colon cancer, and leukemia [62].

The calcium signaling pathway influences the course of cancer, and abnormal calcium signaling pathways can potentially drive tumor cell proliferation, invasion, and metastasis [63]. Furthermore, irregular bile secretion is strongly associated with the progression of malignant tumors like hepatocellular carcinoma and gallbladder cancer [64]. The results of GSEA showed that SPSB2 and Vegfavegfr2 Signaling Pathway, amino acid metabolism, metabolism of amino acids and derivatives, Complement Cascade, Complement System, Complement and Coagulation Cascades, and other signaling pathways are related. The signaling pathway involving vascular endothelial growth factor (VEGF) and its receptor (VEGFR2) is of great significance in multiple cancers’ neovascularization and metastasis processes [65,66,67]. Deviations in amino acid metabolism are crucial factors in cancer development. Many cancers have been linked to disruptions in amino acid metabolism processes [68]. Signaling pathways such as Complement Cascade, Complement System, Complement, and Coagulation Cascades are associated with the immune system and coagulation system in relation to a variety of cancers, immune escape, and thrombosis [69,70]. The findings mentioned above indicate that SPSB2 could be vital in the occurrence and development of LIHC.

Our study examined the relationship between the expression of SPSB2 and 24 different immune cell types in LIHC patients. We found that immune cell infiltration is essential for better patient prognosis. On the contrary, low levels of immune cell infiltration lead to the immune escape of cancer cells, which ultimately results in poor prognosis [71]. The analysis of immune infiltration showed that SPSB2 expression was positively correlated with the infiltration levels of aDCs, NK CD56bright cells, TFH, and Th2 cells. At the same time, SPSB2 expression was negatively correlated with the infiltration level of Eosinophils, Th17 cells, neutrophils, Tcm, DC, Mast cells, CD8 T cells, Treg, and NK cells. During tumor progression, the inhibitory effects of neutrophils and anti-inflammatory responses may be altered, resulting in possible aberrant activation of neutrophils, which promotes tumor cell migration and invasion, stimulates tumor growth and progression, and suppresses immune responses [72]. Moreover, the immunomodulatory interactions between lymphocytes and non-lymphocytes will likely be disrupted. This disruption can result in immune escape, immunosuppression, the acceleration of tumor growth and progression, and the dampening of the effectiveness of tumor immunotherapy [73]. The significance lies in the transition from Th1/Th2 balance to Th2 dominance. Since Th2 cells can inhibit the cellular immune anti-tumor effects, restoring the Th1/Th2 balance is significant for tumor treatment [74,75]. Tregs are typically highly concentrated in the tumor microenvironment. A large number of Tregs is associated with a poor prognosis [76]. Scarcity in the quantity and functional impairments of NK cells contribute to tumor cells’ evasion from immune surveillance [77]. These findings imply that the upregulation of SPSB2 expression can dampen the anti-tumor immune responses in patients with LIHC.

In our research, correlation analysis indicated a positive correlation between the expression of SPSB2 and that of the immune checkpoints TNFRSF4, PDCD1, LAG3, CD47, and CTLA4. TNFRSF4 is crucial in regulating the activation and proliferation processes of T cells. Abnormal TNFRSF4 signaling pathway may lead to T cell dysfunction [78], thus affecting the control of tumors by immune response. PDCD1, also known as PD-1, is a negative immunomodulatory molecule. PD-1 binds to its ligand, PD-L1, and inhibits the activation and effector function of T cells, allowing tumors to evade immune attack [79,80]. LAG3 serves as an inhibitory immune checkpoint molecule. Along with the expression of CD47 and CTLA4, LAG3 can suppress the activation and proliferation of T cells. LAG3 is an inhibitory immune checkpoint molecule that negatively regulates T cell function by inhibiting stimulatory signaling pathways. Abnormal LAG3 activity may lead to the suppression of T cell function [81,82], thereby reducing the ability to mount an immune response to tumors. CD47 inhibits phagocytosis of macrophages by binding to SIRPα receptors [83,84]. Overexpression of CD47 may allow tumor cells to evade clearance by the immune system, thereby promoting tumor development. CTLA4 is also a negative immunomodulatory molecule that inhibits T-cell activation and proliferation upon binding to CD80/86 ligands [85]. Aberrant activation of the CTLA-4 signaling pathway can trigger immunosuppression and impact the body’s immune response to tumors. Our study’s outcomes indicate that SPSB2 is strongly linked to immune cell infiltration and immunosuppression within the tumor microenvironment of LIHC.

Ultimately, our research utilized Huh7 and SMMC-7721 cells to validate the biological function of SPSB2. The expression of SPSB2 was decreased through stable transfection of small interfering RNA (siRNA). Inhibition of SPSB2 expression effectively attenuated the proliferative viability of the two hepatocellular carcinoma cell lines and reduced the cells’ metastatic and invasive abilities. This finding confirmed that SPSB2 is closely connected with tumor progression and implicated in tumor cells’ invasion and metastasis. Consequently, SPSB2 can serve as a molecular target for tumor treatment.

## 5. Conclusions

To sum up, SPSB2 was highly expressed in LIHC, which was strongly associated with patients’ clinical stages and poor prognosis, indicating that SPSB2 might serve as a biomarker for the early diagnosis of LIHC. SPSB2 potentially contributes to tumor formation by regulating immune-related signaling pathways and facilitating immune cell infiltration. In addition, SPSB2 was significantly correlated with TNFRSF4, PDCD1, LAG3, CD47, and CTLA4 immune checkpoints. Consequently, SPSB2 is anticipated to function as a potential biomarker for the diagnosis and prognosis assessment of LIHC. Additionally, it can serve as a novel target for developing antitumor drugs. However, our findings still need further validation, including animal and cellular experiments, to elucidate how SPSB2 promotes tumorigenesis and progression of LIHC.

## Figures and Tables

**Table 1:** SPSB2 expression associated with clinicopathological characteristics (baseline data sheet).

Characteristics	Low Expression of SPSB2	High Expression of SPSB2	*p* Value
n	187	187	
Age, n (%)			0.109
≤60	81 (21.7%)	96 (25.7%)	
>60	106 (28.4%)	90 (24.1%)	
Weight, n (%)			<0.001
≤70	72 (20.8%)	112 (32.4%)	
>70	101 (29.2%)	61 (17.6%)	
Gender, n (%)			0.320
Female	65 (17.4%)	56 (15%)	
Male	122 (32.6%)	131 (35%)	
Race, n (%)			<0.001
Asian	62 (17.1%)	98 (27.1%)	
Black or African American	7 (1.9%)	10 (2.8%)	
White	112 (30.9%)	73 (20.2%)	
Pathologic T stage, n (%)			0.017
T1	106 (28.6%)	77 (20.8%)	
T2	38 (10.2%)	57 (15.4%)	
T3	34 (9.2%)	46 (12.4%)	
T4	6 (1.6%)	7 (1.9%)	
Pathologic N stage, n (%)			0.094
N0	115 (44.6%)	139 (53.9%)	
N1	4 (1.6%)	0 (0%)	
Pathologic M stage, n (%)			0.710
M0	126 (46.3%)	142 (52.2%)	
M1	1 (0.4%)	3 (1.1%)	
Pathologic stage, n (%)			0.041
Stage I	98 (28%)	75 (21.4%)	
Stage II	34 (9.7%)	53 (15.1%)	
Stage III	38 (10.9%)	47 (13.4%)	
Stage IV	2 (0.6%)	3 (0.9%)	
Tumor status, n (%)			0.312
Tumor free	106 (29.9%)	96 (27%)	
With tumor	72 (20.3%)	81 (22.8%)	
Histological type, n (%)			0.117
Hepatocellular carcinoma	182 (48.7%)	182 (48.7%)	
Fibrolamellar carcinoma	3 (0.8%)	0 (0%)	
Hepatocholangiocarcinoma (mixed)	2 (0.5%)	5 (1.3%)	
BMI, n (%)			<0.001
≤25	68 (20.2%)	109 (32.3%)	
>25	98 (29.1%)	62 (18.4%)	
Residual tumor, n (%)			0.970
R0	162 (47%)	165 (47.8%)	
R1&R2	9 (2.6%)	9 (2.6%)	
Histologic grade, n (%)			<0.001
G1	33 (8.9%)	22 (6%)	
G2	105 (28.5%)	73 (19.8%)	
G3	42 (11.4%)	82 (22.2%)	
G4	2 (0.5%)	10 (2.7%)	
AFP(ng/mL), n (%)			0.024
≤400	117 (41.8%)	98 (35%)	
>400	25 (8.9%)	40 (14.3%)	
Albumin(g/dL), n (%)			0.908
<3.5	35 (11.7%)	34 (11.3%)	
≥3.5	119 (39.7%)	112 (37.3%)	
Prothrombin time, n (%)			0.281
≤4	105 (35.4%)	103 (34.7%)	
>4	51 (17.2%)	38 (12.8%)	
Child-Pugh grade, n (%)			0.200
A	108 (44.8%)	111 (46.1%)	
B&C	14 (5.8%)	8 (3.3%)	
Fibrosis ishak score, n (%)			0.695
0	44 (20.5%)	31 (14.4%)	
1/2	14 (6.5%)	17 (7.9%)	
3/4	14 (6.5%)	14 (6.5%)	
5	5 (2.3%)	4 (1.9%)	
6	42 (19.5%)	30 (14%)	
Adjacent hepatic tissue inflammation, n (%)			0.164
None	72 (30.4%)	46 (19.4%)	
Mild	49 (20.7%)	52 (21.9%)	
Severe	9 (3.8%)	9 (3.8%)	

**Table 2:** Results of Cox regression analysis of different clinicopathological factors and expression values of SPSB2.

Characteristics	Total (N)	Univariate Analysis		Multivariate Analysis	
		Hazard Ratio (95% CI)	*p* Value	Hazard Ratio (95% CI)	*p* Value
Gender	373				
Female	121	Reference			
Male	252	0.793 (0.557–1.130)	0.200		
Race	361				
Asian	159	Reference			
Black or African American	17	1.585 (0.675–3.725)	0.290		
White	185	1.323 (0.909–1.928)	0.144		
Age	373				
≤60	177	Reference			
>60	196	1.205 (0.850–1.708)	0.295		
Weight	345				
≤70	184	Reference			
>70	161	0.941 (0.657–1.346)	0.738		
BMI	336				
≤25	177	Reference			
>25	159	0.798 (0.550–1.158)	0.235		
Adjacent hepatic tissue inflammation	236				
None	118	Reference			
Mild	101	1.204 (0.723–2.007)	0.476		
Severe	17	1.144 (0.447–2.930)	0.779		
Pathologic T stage	370				
T1&T2	277	Reference		Reference	
T3	80	2.355 (1.618–3.430)	<0.001	2.439 (1.551–3.836)	<0.001
T4	13	4.733 (2.424–9.241)	<0.001	4.753 (1.769–12.771)	0.002
Pathologic N stage	258				
N0	254	Reference			
N1	4	2.029 (0.497–8.281)	0.324		
Pathologic M stage	272				
M0	268	Reference		Reference	
M1	4	4.077 (1.281–12.973)	0.017	0.946 (0.224–3.996)	0.940
Residual tumor	344				
R0	326	Reference			
R1&R2	18	1.604 (0.812–3.169)	0.174		
Histologic grade	368				
G1	55	Reference			
G2	178	1.162 (0.686–1.969)	0.576		
G3	123	1.185 (0.683–2.057)	0.545		
G4	12	1.681 (0.621–4.549)	0.307		
AFP(ng/mL)	279				
≤400	215	Reference			
>400	64	1.075 (0.658–1.759)	0.772		
Albumin(g/dL)	299				
<3.5	69	Reference			
≥3.5	230	0.897 (0.549–1.464)	0.662		
Prothrombin time	296				
≤4	207	Reference			
>4	89	1.335 (0.881–2.023)	0.174		
Vascular invasion	317				
No	208	Reference			
Yes	109	1.344 (0.887–2.035)	0.163		
Fibrosis ishak score	214				
0	75	Reference			
1/2	31	0.936 (0.437–2.002)	0.864		
3/4	28	0.699 (0.288–1.695)	0.428		
5	9	0.764 (0.181–3.228)	0.714		
6	71	0.734 (0.401–1.345)	0.317		
SPSB2	373				
Low	187	Reference		Reference	
High	186	1.781 (1.253–2.534)	0.001	1.965 (1.234–3.129)	0.004

**Table 3:** GO analysis.

Ontology	ID	Description	GeneRatio	BgRatio	*p* Value	p.adjust	Zscore
BP	GO:0010817	Regulation of hormone levels	121/2389	496/18,800	4.48 × 10^−13^	2.62 × 10^−9^	0.2727273
BP	GO:0023061	Signal release	107/2389	451/18,800	6.18 × 10^−11^	9.59 × 10^−8^	3.7702723
BP	GO:0071466	Cellular response to xenobiotic stimulus	52/2389	168/18,800	3.95 × 10^−10^	3.84 × 10^−7^	0.2773501
BP	GO:0006805	Xenobiotic metabolic process	36/2389	108/18,800	2.25 × 10^−8^	5.95 × 10^−6^	−0.3333333
BP	GO:0007389	Pattern specification process	98/2389	463/18,800	1.82 × 10^−7^	3.67 × 10^−5^	7.4751288
CC	GO:0062023	Collagen-containing extracellular matrix	111/2532	429/19,594	2.17 × 10^−13^	1.34 × 10^−10^	−0.8542422
CC	GO:0009925	Basal plasma membrane	64/2532	251/19,594	4.74 × 10^−8^	3.25 × 10^−6^	2.0000000
CC	GO:0071162	CMG complex	8/2532	11/19,594	8.83 × 10^−6^	0.0002	2.8284271
CC	GO:0005604	Basement membrane	26/2532	95/19,594	0.0001	0.0023	1.5689291
CC	GO:0005581	Collagen trimer	23/2532	86/19,594	0.0004	0.0068	−0.2085144
MF	GO:0030546	Signaling receptor activator activity	134/2437	496/18,410	1.02 × 10^−16^	1.08 × 10^−13^	4.1465684
MF	GO:0048018	Receptor ligand activity	131/2437	489/18,410	4.7 × 10^−16^	2.49 × 10^−13^	4.4558907
MF	GO:0046873	metal ion Transmembrane transporter activity	107/2437	428/18,410	2.53 × 10^−11^	8.93 × 10^−9^	4.7370088
MF	GO:0008083	Growth factor activity	50/2437	162/18,410	3.85 × 10^−9^	5.1 × 10^−7^	0.5656854
MF	GO:0005125	Cytokine activity	62/2437	235/18,410	4.75 × 10^−8^	4.58 × 10^−6^	2.7940028

Note: BP, Biological process; CC, cellular component; MF, molecular function.

**Table 4:** KEGG analysis.

Ontology	ID	Description	GeneRatio	BgRatio	*p* Value	p.adjust	Zscore
KEGG	hsa04080	Neuroactive ligand-receptor interaction	111/1118	362/8164	8.99 × 10^−18^	2.94 × 10^−15^	4.0813794
KEGG	hsa00982	Drug metabolism—cytochrome P450	27/1118	72/8164	3.47 × 10^−7^	3 × 10^−5^	−1.7320508
KEGG	hsa04976	Bile secretion	27/1118	89/8164	3.45 × 10^−5^	0.0014	−1.3471506
KEGG	hsa00250	Alanine, aspartate and glutamate metabolism	15/1118	37/8164	4.96 × 10^−5^	0.0016	−1.2909944
KEGG	hsa04020	Calcium signaling pathway	55/1118	240/8164	5.82 × 10^−5^	0.0017	1.4832397
KEGG	hsa05204	Chemical carcinogenesis—DNA adducts	22/1118	69/8164	7.9 × 10^−5^	0.0019	−2.1320072
KEGG	hsa04512	ECM-receptor interaction	25/1118	88/8164	0.0002	0.0044	1.8000000
KEGG	hsa04110	Cell cycle	32/1118	126/8164	0.0003	0.0052	4.9497475
KEGG	hsa00040	Pentose and glucuronate interconversions	12/1118	35/8164	0.0016	0.0206	−1.1547005
KEGG	hsa00983	Drug metabolism—other enzymes	21/1118	80/8164	0.0020	0.0235	−0.6546537

Note: KEGG, Kyoto Encyclopedia of Genes and Genomes.

## Data Availability

The datasets generated and/or analyzed during the current study are available in the GEO (https://www.ncbi.nlm.nih.gov/geo/ accessed on 7 October 2024), UCSC XENA (https://xenabrowser.net/datapages/ accessed on 5 October 2024), TCGA (https://portal.gdc.cancer.gov, accessed on 7 October 2024), and HPA (https://www.proteinatlas.org/ accessed on 15 October 2024) repositories. The datasets generated during and/or analyzed during the current study are also available from the corresponding author upon reasonable request.

## Abbreviations

LIHC	Liver Hepatocellular Carcinoma
TCGA	The Cancer Genome Atlas
GEO	Gene Expression Omnibus
GO	Gene Ontology
KEGG	Kyoto Encyclopedia of Genes and Genomes
GSEA	Gene set enrichment analysis
OS	Overall survival
ANOVA	One-factor analysis of variance
